# UV-Pre-Treated and Protein-Adsorbed Titanium Implants Exhibit Enhanced Osteoconductivity

**DOI:** 10.3390/ijms21124194

**Published:** 2020-06-12

**Authors:** Yoshihiko Sugita, Juri Saruta, Takashi Taniyama, Hiroaki Kitajima, Makoto Hirota, Takayuki Ikeda, Takahiro Ogawa

**Affiliations:** 1 Weintraub Center for Reconstructive Biotechnology, Division of Advanced Prosthodontics, UCLA School of Dentistry, Los Angeles, CA 90095-1668, USA; yosshii@dpc.agu.ac.jp (Y.S.); taniyama.orth@tmd.ac.jp (T.T.); hiroaki_k_0315@yahoo.co.jp (H.K.); mhirota@yokohama-cu.ac.jp (M.H.); ikeda.takayuki@nihon-u.ac.jp (T.I.); togawa@dentistry.ucla.edu (T.O.); 2 Department of Oral Pathology, School of Dentistry, Aichi Gakuin University, 1-1-100 Kusumoto-cho, Chikusa-ku, Aichi 464-8650, Japan; 3 Department of Oral Science, Graduate School of Dentistry, Kanagawa Dental University, 82 Inaoka, Kanagawa 238-8580, Japan; 4 Department of Oral and Maxillofacial Surgery, Graduate School of Medicine, Yokohama City University, 3-9 Fukuura, Kanazawa-ku, Kanagawa 236-0004, Japan; 5 Department of Oral and Maxillofacial Surgery/Orthodontics, Yokohama City University Medical Center, 4-57 Urafune-cho, Kanagawa 232-0024, Japan

**Keywords:** titanium implants, osteoblast, fibronectin, UV-photofunctionalization, bone morphogenetic protein-2, mechanical anchorage, osteoconductivity

## Abstract

Titanium materials are essential treatment modalities in the medical field and serve as a tissue engineering scaffold and coating material for medical devices. Thus, there is a significant demand to improve the bioactivity of titanium for therapeutic and experimental purposes. We showed that ultraviolet light (UV)-pre-treatment changed the protein-adsorption ability and subsequent osteoconductivity of titanium. Fibronectin (FN) adsorption on UV-treated titanium was 20% and 30% greater after 1-min and 1-h incubation, respectively, than that of control titanium. After 3-h incubation, FN adsorption on UV-treated titanium remained 30% higher than that on the control. Osteoblasts were cultured on titanium disks after 1-h FN adsorption with or without UV-pre-treatment and on titanium disks without FN adsorption. The number of attached osteoblasts during the early stage of culture was 80% greater on UV-treated and FN-adsorbed (UV/FN) titanium than on FN-adsorbed (FN) titanium; osteoblasts attachment on UV/FN titanium was 2.6- and 2.1-fold greater than that on control- and UV-treated titanium, respectively. The alkaline phosphatase activity of osteoblasts on UV/FN titanium was increased 1.8-, 1.8-, and 2.4-fold compared with that on FN-adsorbed, UV-treated, and control titanium, respectively. The UV/FN implants exhibited 25% and 150% greater in vivo biomechanical strength of bone integration than the FN- and control implants, respectively. Bone morphogenetic protein-2 (BMP-2) adsorption on UV-treated titanium was 4.5-fold greater than that on control titanium after 1-min incubation, resulting in a 4-fold increase in osteoblast attachment. Thus, UV-pre-treatment of titanium accelerated its protein adsorptivity and osteoconductivity, providing a novel strategy for enhancing its bioactivity.

## 1. Introduction

Titanium has excellent mechanical strength, corrosion resistance, and biocompatibility [1]. Titanium materials have been widely used in the medical field and are modalities of requisite treatment in bone and teeth restorative surgeries [2,3,4]. Additionally, titanium is used as a tissue engineering scaffold and cover-up material for diverse medical devices. Thus, there is a marked demand to modify the biological activity for the purposes of clinical application and development of novel biomaterial [5,6].

Ultraviolet (UV) light photofunctionalization involves the treatment of titanium implant surfaces with UV light immediately before use and is a well-known and effective technique for overcoming the biological aging of titanium [7,8]. The optimal effect of photofunctionalization is achieved via three mechanisms: removal of hydrocarbons from the implant surface, conversion of surface hydrophobicity to hydrophilicity, and conversion of the surface charge from negative to positive [7,9,10,11]. Therefore, the definition of UV photofunctionalization is a modification of titanium surface after UV irradiation, including change in its physicochemical surface properties and upregulating its biological capability [12]. Furthermore, UV treatment improves the mechanical anchorage of dental implants and is, therefore, of great value in both basic and clinical research [13,14,15,16]. The bone to implant integration capability was increased more than 3 times compared with untreated implants after a 2-weeks healing period in an animal model. The UV-treated implants showed almost 100% between bone and implant contact, whereas this value for untreated implants was 55% at week 4 of healing time [9]. Funato et al. reported the clinical prognosis of UV-treated dental implants [17]. The healing time was approximately 3 months in UV-treated implants, and approximately 6 months in control implants before functional loading, accelerated healing observed in the former. Moreover, the monthly increase in the implant stability quotient, a clinical measure that quantifies the stability of dental implants, ranged from 2.0 to 8.7 in UV-photofunctionalized implants, which was considerably higher than that of control implants [17].

UV treatment on the titanium surface enhances hydrophilicity and removes hydrocarbons. However, it does not change the morphology of the titanium surface. This alteration of the surface is critical for cell proliferation, differentiation, and bone integration capability [18,19,20]. It is important to have evidence from both in vitro and in vivo results, to help understand the underlying biological processes and mechanisms. The attachment and adhesion of osteoblasts are enhanced by the hydrophilicity on the titanium surface. The hydrocarbons deposited on the titanium surface after UV treatment are removed, thereby promoting their further cellular phenotype [21,22]. Depending on the quantity of hydrocarbons on the titanium surface, they adversely affect osteoblast’s activity [9]. On hydrocarbon-enriched titanium surfaces, osteoblasts appear rounded, their cytoskeleton formation appears suppressed, and cellular proliferation is delayed [23].

Titanium has corrosion resistance and is used as bioinert implantable materials [1,24]. Therefore, titanium surfaces do not actively interact with cells. In fact, it is well-known that titanium surfaces require ionic bridges (particularly formed by divalent cations) to attract cells [7,25]. Ordinary titanium surfaces are electronegative in nature, as are cells [7]. Therefore, titanium surfaces attract cells only in the presence of divalent cations, such as Ca^2+^ [26,27]. However, UV-treated titanium surfaces are positively charged and act as chemoattractants that aggressively attract cells necessary for bone-implant integration, without the aid of divalent cations [7].

To further advance titanium implant therapy with stronger and faster bone-implant integration, we attempted to develop a protein-coating technique distinct from similar techniques reported previously. Specifically, we hypothesized that UV-treatment of titanium enhances and accelerates protein coating on titanium, which potentially can be applied immediately prior to implant surgery as a synergetic activation technique for implants. The objective of this study was to investigate the biological activity and bone-titanium integration capability of protein-coated titanium with or without UV-pre-treatment.

## 2. Results

### 2.1. Characteristics of Titanium Disks Surface with or without UV Treatment

**Figure 1:**
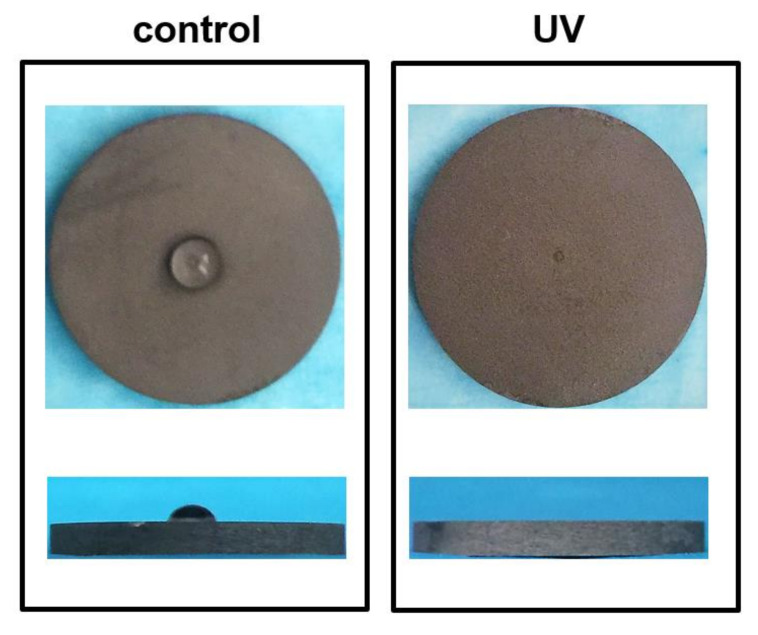
Hydrophobicity/hydrophilicity status of titanium surfaces. Top- and side view images of 10 µL double-distilled H_2_O placed on titanium disks. The control titanium surface showed hydrophobic status, whereas the UV-treated surface showed superhydrophilic status.

The double-distilled water was dropped on the surface of the control and UV-treated titanium disks carefully, and the water and the surface contact angles were measured (Figure 1). The water dropped on the UV-treated titanium surface spread immediately after dripping. The surface of the control surfaces was hydrophobic, but the UV-treated surface showed super hydrophilicity and was similar to the results of other published data [13]. The ratio that the control and UV-treated titanium surface were covered with an atomic of carbon was shown to be 42% and 15% by x-ray photoelectron spectroscopy (XPS).

### 2.2. Enhanced Protein Adsorption on UV-Treated Titanium

**Figure 2:**
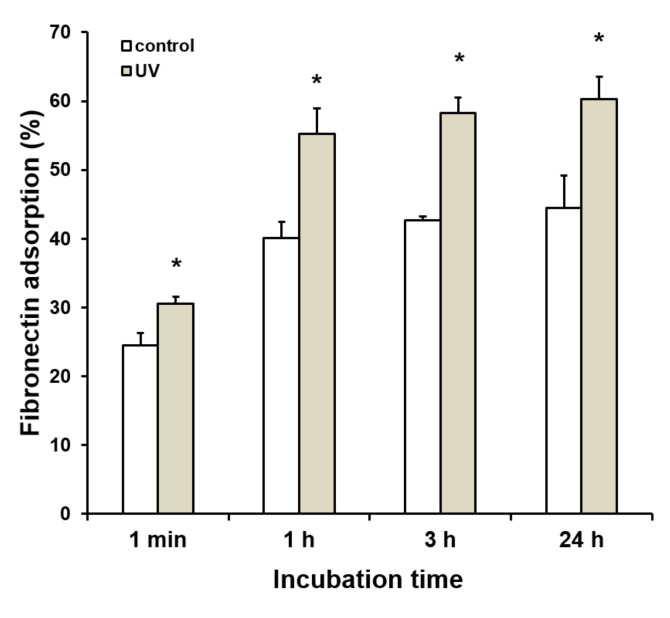
Enhanced protein adsorption on titanium surfaces using UV treatment. Data represent means ± standard deviation of adsorption rates of bovine plasma fibronectin (FN) after 1 min and 1, 3, and 24 h of incubation for control- and UV-treated titanium disks (*n* = 3). Asterisks indicate significant differences (*p < 0.05, Welch’s *t*-test).

After incubation for 1 min and 1, 3, and 24 h, fibronectin (FN) adsorption was evaluated on the control and UV-treated titanium surface. The FN adsorption showed a greater extent in the UV-treated surface than control of all time-points. In both control and UV-treated titanium surface, the FN adsorption was a plateau after 3 h incubation, but the extent of adsorption on the UV-treated titanium was 30% higher than that on the control surface (Figure 2).

### 2.3. Enhanced Initial Cell Attachment to UV-Treated and Fibronectin-Adsorbed Titanium

**Figure 3:**
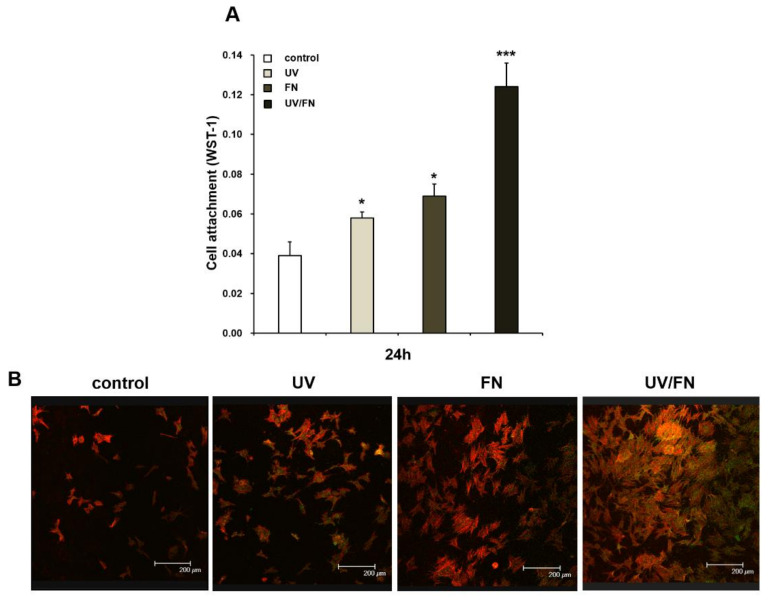
Attachment of rat bone marrow-derived osteoblasts on control, UV-treated (UV), fibronectin-adsorbed (FN), and UV-treated and fibronectin-adsorbed (UV/FN) titanium surfaces. A 1-h adsorption protocol was used in this experiment. (**A**) The number of attached cells after 24-h incubation was evaluated by water soluble tetrazolium salts-1 (WST-1) assay. (**B**) Microscopic appearance. The initial spread of osteoblasts at 24 h after seeding on control, UV, FN, and UV/FN titanium surfaces. Representative confocal microscopy images of cells stained with rhodamine-phalloidin, for actin filaments (red), and anti-vinculin, for vinculin (green). Each value represents the mean ± standard deviation from triplicate experiments (*n* = 3). *p < 0.05 and ***p < 0.001 represent significant differences relative to the control surface. Scale bar = 200 µm.

The initial attached number of cells on the different titanium surfaces was evaluated using water soluble tetrazolium salts-1 (WST-1) 24 h of culture (Figure 3A). In this experiment, a 1 h adsorption method to adsorb was used. There was a significantly higher number of cells, which attached to the UV-treated (UV), FN-adsorbed (FN), and UV-treated and FN-adsorbed (UV/FN) disks, than the control surface. Notably, the number of osteoblasts was 80% greater on the UV/FN disk than on the FN disk. Thus, the initial cell attachment was enhanced by a combination of UV treatment and FN adsorption at least three times compared to the control titanium surfaces. This effect was confirmed using the image by Confocal microscopic low-magnification of the actin filaments of osteoblast stained with rhodamine-phalloidin (Figure 3B).

### 2.4. Enhanced Cell Spreading on UV-Treated and Fibronectin-Adsorbed Titanium Surface

**Figure 4:**
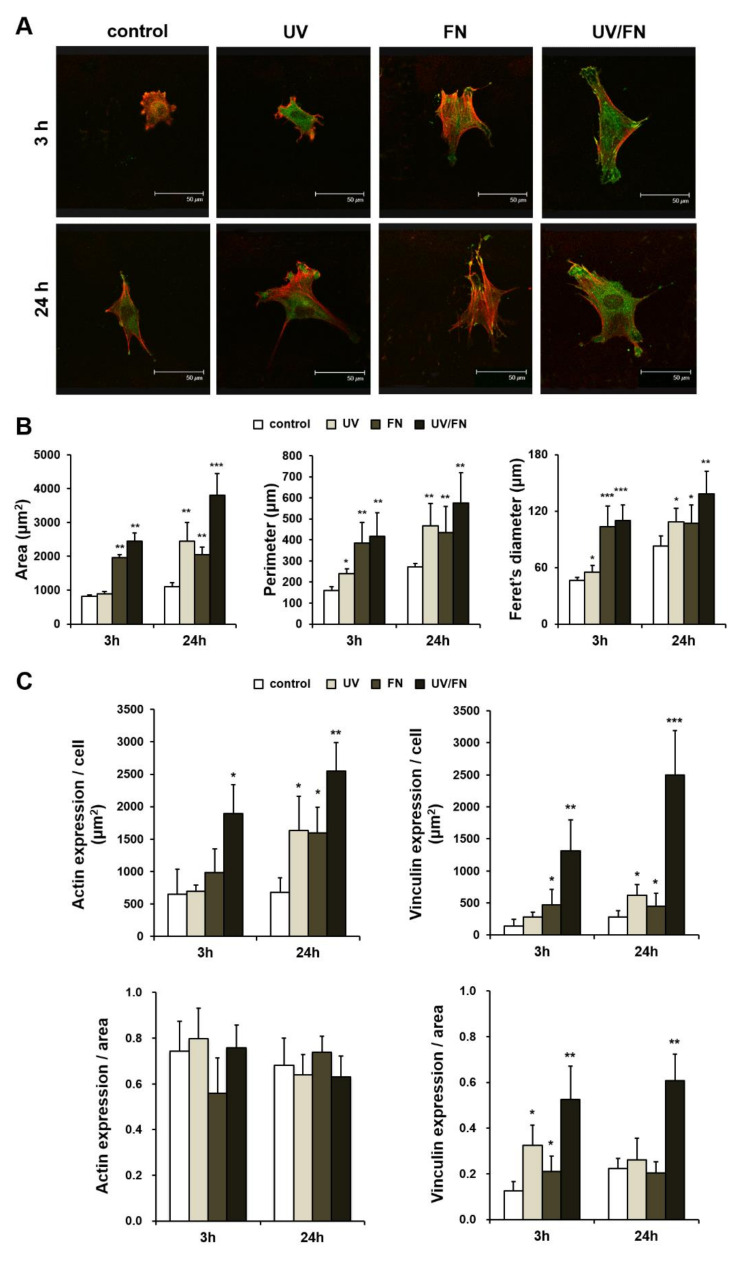
Representative confocal microscopy images of the attachment and spreading behavior of osteoblasts after 3 and 24 h of seeding on the control, UV-treated (UV), fibronectin-adsorbed (FN), and UV-treated and fibronectin-adsorbed (UV/FN) titanium surfaces. A 1-h adsorption protocol was used in this experiment. (**A**) Initial attachment, spread, cytoskeletal arrangement, and establishment of focal adhesion of osteoblasts after 3 and 24 h of seeding onto the titanium surfaces. Confocal microscopy images of osteoblasts following immunochemical staining for cytoskeletal actin (red) and adhesion protein vinculin (green) are shown. Scale bar = 50 µm. (**B**) Histograms for cytomorphometric parameters (cell area, cell perimeter, and Feret’s diameter) measured from the images. (**C**) Expression levels of actin and vinculin were semi-quantified using confocal microscopy images. Data are the means ± standard deviation from triplicate experiments (*n* = 8). *p < 0.05, **p < 0.01, and ***p < 0.001 represent significant differences relative to the control surface at each time-point.

The double-stained cells were observed by CLSM to confirm their adhesion and cell morphology on the control-, UV-, FN-, and UV/FN-treated titanium surfaces in both 3- and 24-h of culture. A 1-h adsorption protocol was used in this experiment (Figure 4A). After 3 h and 24 h incubation, CLSM of rhodamine phalloidin-stained osteoblasts was larger on UV- and FN-treated surfaces compared to the control surface. The cells on the UV-treated surface observed the spread of polytropic actin projection, but the cells on the control surface were rounded, and the projected cell processes were neither observed nor exhibited development of cytoskeleton. Furthermore, the expression of vinculin in the cells on the UV-treated titanium surface was stronger and more spread than the cells on the control surface. At the early stage of culture, the vinculin-positive area was expressed in stretching tips of projection of the cells on UV-treated titanium surfaces. After 24 h incubation, the cells of control and UV-treated surface were larger than the cells incubated for 3 h. Additionally, the UV-treated surfaces showed larger cells with greater numbers of cell processes; the cells exhibited more strongly and mature development of cytoskeleton. At 3-h, the adhesion protein was observed in localization, especially for the development of the cell projection in the cultured cells on the UV-treated surfaces (Figure 4A). The cell area, perimeter, and Feret’s diameter of the cells were demonstrated by cytomorphometry (Figure 4B). The expression of actin and vinculin were more strongly observed in the majority of cells on UV-treated surfaces. At both 3 and 24 h of incubation, no significant difference was observed among different surfaces regarding the actin expression/area ratio, but the vinculin expression/area ratio was highest on the UV/FN-treated titanium surface (Figure 4C).

### 2.5. Enhanced Osteoblastic Phenotype on UV-Treated and Fibronectin-Adsorbed Titanium

**Figure 5:**
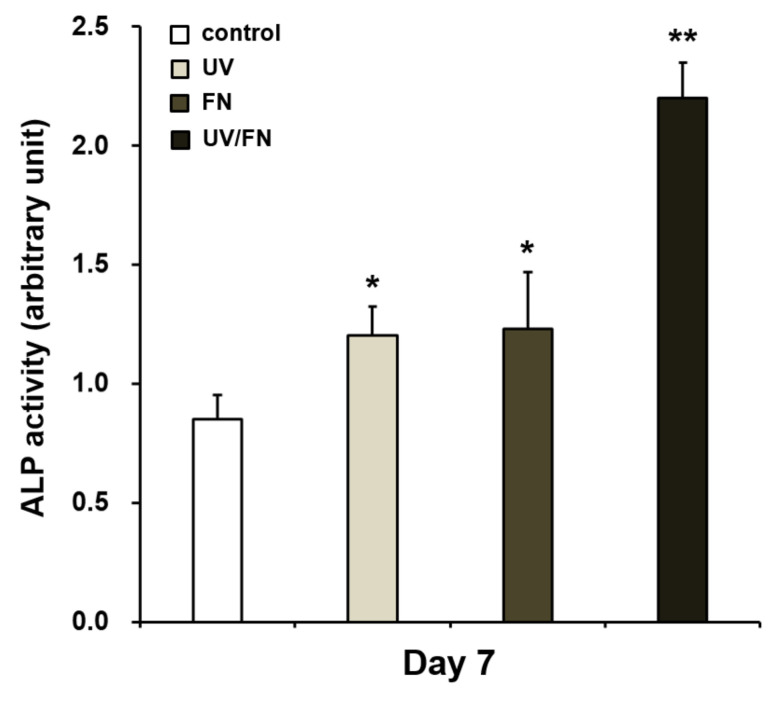
UV treatment enhanced the activity of alkaline phosphatase (ALP), a marker of early- to middle-stage osteoblast differentiation, on control, UV-treated (UV), fibronectin-adsorbed (FN), and UV-treated and fibronectin-adsorbed (UV/FN) titanium surfaces. A 1-h adsorption protocol was used in this experiment. ALP activity on day 7 was quantified by colorimetry and standardized relative to cell number. Each value represents the mean ± standard deviation of triplicate experiments (*n* = 3). *p < 0.05 and **p < 0.01 represent significant differences relative to the control surface.

The differentiation of osteoblast was assayed using the alkaline phosphatase (ALP) activity assay. UV treatment enhanced the osteoblastic phenotype on titanium surfaces. The expression of ALP—a marker of the differentiation of osteoblast in the middle from the early stage—was considerably increased in comparison with the control surface on UV-treated- and FN surfaces. This effect was most pronounced on the UV/FN-treated titanium surface (Figure 5).

### 2.6. Combination of UV Treatment and FN Adsorption Enhanced Mechanical Anchorage

**Figure 6:**
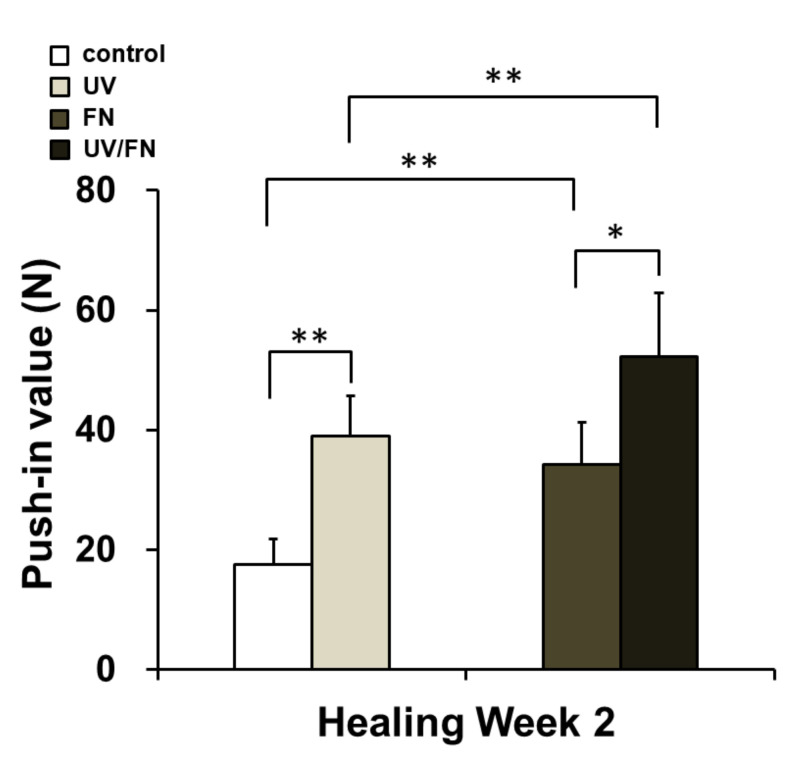
In vivo bone integration ability of differently treated titanium implants. The biomechanical strength of bone-implant integration was evaluated using the biomechanical push-in test. Implants with control, UV-treated (UV), fibronectin-adsorbed (FN), and UV-treated and fibronectin-adsorbed (UV/FN) titanium surfaces were compared. A 1-h adsorption protocol was used in this experiment. Each bar represents the mean ± standard deviation of the control, UV, FN, and UV/FN implant groups (each *n* = 6; healing time: 2 weeks). *p < 0.05 and **p < 0.01 represent significant differences as determined by two-way ANOVA followed by Bonferroni test.

A push- in value was used to evaluate mechanical anchorage in the control, UV, FN, and UV/FN implants (Figure 6). The average of push-in value for UV-treated implants was about two times higher than that of the control implants. Notably, the push-in values of UV-treated implants and the FN-adsorbed implants were equivalent. Furthermore, the strength of bone-implant integration of the UV/FN implants was significantly higher than that of the FN-adsorbed implants, indicating that the most effective increase was observed in the combination of UV treatment and FN adsorption as a mechanical anchorage.

### 2.7. Tissue Morphology and Chemistry around Implants

**Figure 7:**
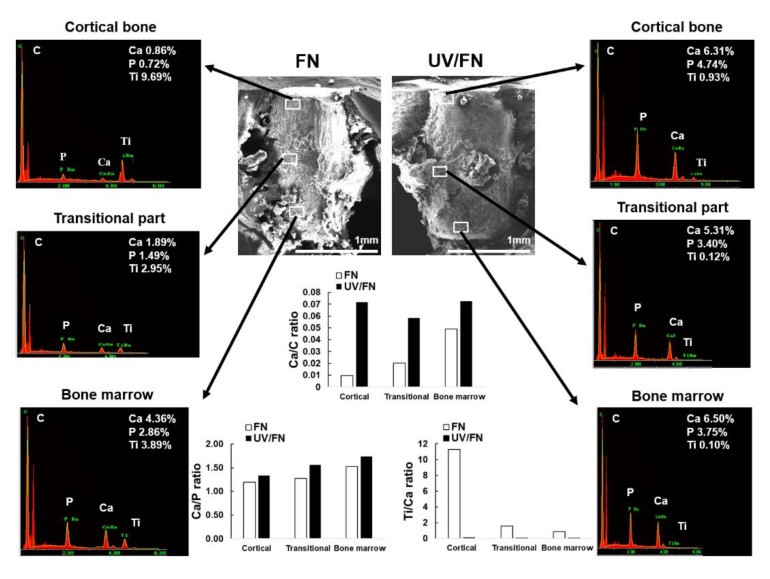
Morphology and elemental analyses of fibronectin-adsorbed (FN) and UV-treated and fibronectin-adsorbed (UV/FN) implant interfaces at the early healing stage of week two. A 1-h adsorption protocol was used. Implants were retrieved after the push-in test; the tissue interfaces were exposed and analyzed by scanning electron microscopy (SEM) and energy-dispersive X-ray spectroscopy (EDS). FN-adsorbed and UV/FN-treated implants were compared. Third zones of the implant from each of the superior (cortical), mid (transitional), and apical (bone marrow) areas are presented with the EDS spectrum obtained from the respective areas. The arrows show the results of the EDS analysis in those areas. Calculated Ca/C, Ca/P, and Ti/Ca ratios are also presented. Scale bar = 1 mm.

FN- and UV/FN-treated implants isolated from rat femurs were investigated morphologically and chemically using an energy-dispersive X-ray spectroscopy (EDS) spectrum. Representative images of specimens of 2 weeks are shown in Figure 7. SEM images of 2 weeks implants showed that the UV/FN surfaces were more extensively covered with biological tissue than the FN-adsorbed implants. Almost all areas of the UV/FN implants were covered by tissue and showed higher Ca and P peaks compared to the FN-adsorbed implants. A weak Ti signal was detected in almost all areas of the UV/FN implants. These indicated that the calcified tissue had sufficient thickness and/or quality to mask the titanium surface. In contrast, although the bone marrow zone of the FN-adsorbed implant surface was covered with tissue, as indicated by the obvious Ca and P peaks in the EDS spectrum, mineralized tissue was sparsely observed in the cortical zone and mid-zone areas. The Ti/Ca ratio was remarkably higher in the FN-adsorbed implants than in the UV/FN-treated implants. The Ca/C ratio was considerably higher in the UV/FN implants, demonstrating that it was more completely covered with calcified tissue on the UV/FN implants than on the FN-adsorbed implants in all three zones. The Ca/P ratio was slightly higher in the UV/FN implants than in the FN-adsorbed implants in the three zones.

### 2.8. UV-Enhanced Bone Morphogenetic Protein-2 (BMP-2) Adsorption to Titanium

**Figure 8:**
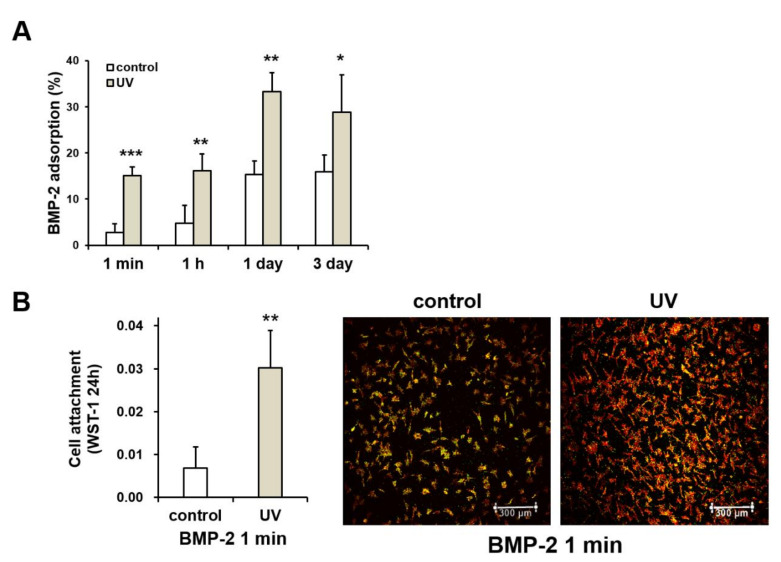
Effect of UV treatment on bone morphogenetic protein-2 (BMP-2) adsorption ability of titanium. (**A**) Adsorption rates (means ± standard deviation) of bovine plasma BMP-2 after 1 min, 1 h, 1 day, and 3 days of incubation on control and UV-treated (UV) surfaces (*n* = 3). (**B**) Initial cellular attachment to titanium surfaces. A 1-min adsorption protocol was used. Rat bone marrow-derived osteoblasts attached to the control and UV-treated titanium disks were evaluated by WST-1 assay after 24 h of seeding. Fluorescence micrographs of osteoblasts cultured on control- and UV-treated titanium surfaces after 24 h of seeding. Representative images of cells stained with rhodamine-phalloidin for actin (red: cytoskeleton protein) and an antibody to vinculin (green: focal adhesion-related protein) are presented. Scale bar = 300 µm. Each value represents the mean ± standard deviation from triplicate experiments (*n* = 3). Asterisks indicate significant differences (*p < 0.05, **p < 0.01, ***p < 0.001, Welch’s *t*-test).

The extent of adsorption of bone morphogenetic protein-2 (BMP-2) was examined on the control and UV-treated titanium surfaces at different incubation time points. The UV-treated surfaces showed higher BMP-2 adsorption than the control surfaces at all time-points (Figure 8A). At 24 h after seeding, attachment of osteoblasts derived from rat bone marrow was examined by means of titanium disks on the titanium surfaces. A 1-min BMP-2 adsorption protocol was used in this experiment. The number of attached osteoblasts to the UV-treated surfaces was significantly higher than those of cells to control surfaces (Figure 8B). The images provided by low-magnification confocal microscopy supported these quantitative data (Figure 8B).

## 3. Discussion

In this study, we showed enhancement in the biological properties and increase in osteoconductivity for UV-photofunctionalized protein-coated titanium used as an implant material. The UV/FN titanium surface provided more biological functions than the control. In addition, the protein adsorption on the UV-pre-treated titanium surfaces significantly accelerated and augmented biological functions as opposed to the titanium surfaces that were only UV-treated or FN-adsorbed.

The surface modifications obtained by UV-mediated photofunctionalization and protein coating play an important role in improving the surface characteristics of titanium. Titanium constantly absorbs hydrocarbons and other organic impurities from the environment [28]. The exposed surface exhibits an inverse correlation in the relationship between surface carbon and hydrophilicity [23]. UV treatment removes hydrocarbons and enhances wettability and water adsorption by exciting electrons from the valence band to the conduction band. Oxygen vacancies are formed because of the reduction of Ti^4+^ to Ti^3+^; however, wettability is not significantly correlated with protein adsorption [7,9]. Furthermore, it is an important phenomenon that the activity of osteoblasts is inversely correlated with the state of hydrocarbon contamination on titanium surfaces [23]. Our results also indicated the hydrophilic nature of the titanium surfaces, following UV treatment. Therefore, this physicochemical surface modification was considered as one of the factors that greatly improved osteoblast activity and osteoconductivity in our study.

The enhancement of osteoblastic activity may be attributed to the improved protein adsorption on titanium surfaces. Protein adsorption is known to be affected by various surface properties, such as roughness, organic impurities, chemical composition, van der Waals interactions, and hydrogen bonding [29,30]. Although many reports have described protein coating treatments of implant surfaces in the last decade, most titanium surface treatments require a period of several hours to several days [31,32,33]. However, in the present study, we performed UV pre-treatment and BMP-2 treatment for 1 min or UV pre-treatment and FN treatment for 1 h on the titanium surface for a short period of time using a simple method. The cells clearly showed accelerated and augmented biological activity with short-term pre-treatment using the simple method. Photofunctionalization plays an important role in protein adsorption [9,34]. This process increased FN- and BMP-2 adsorption on the acid-etched titanium surfaces used in this study, although the extent of and time required for adsorption varied depending on the type of FN- and BMP-2 proteins. Furthermore, protein adsorption and cellular attachment are prerequisites for initiating a sequence of osteogenic processes in material for the living body [12,35]. For instance, cell-surface receptors and integrins interact with the proteins adsorbed onto the surface material by binding their amino acid sequence, RGD. This process is important for cell attachment and subsequently regulates cell proliferation and functions [26,27,35]. We observed similar results in our study for protein adsorption and cellular attachment; protein adsorption and osteoblast attachment on UV-treated surfaces increased relative to that on control surfaces. Therefore, the cell spreading behavior was delayed on control surfaces, whereas it was promoted on FN-adsorbed, UV-treated, or UV/FN surfaces. The development and growth of a cellular skeleton and cellular expansion processes are necessary for initiating cellular functions and intracellular signaling, which considerably differed between the UV/FN and control titanium surfaces.

The integration strength results showed that the strength significantly increased on the UV/FN surfaces compared to that on the titanium surface, which was only FN-adsorbed using an in vivo push-in test. Moreover, the consistent biological effects of surface differences from cortical bone zone to bone marrow zone at a 2-week healing period were demonstrated by interfacial analyses. The SEM images and EDS elemental spectra of the UV/FN implants suggested that bone creation on this surface was more extensive than that observed on FN-adsorbed implants, supporting the observed increased push-in value. Ti signals were significantly masked in the UV/FN implants in three zones of the cortical bone, transitional part, and bone marrow as compared to that in the FN-coated implants. This indicated that the mineralized tissues on the UV/FN implants were thicker, more mature, or both. Moreover, the higher Ca/C and Ca/P ratios for the UV/FN implants in the cortical bone, transitional part, and bone marrow zone suggested that the quality of osteogenesis improved after photofunctionalization. The in vitro culture conditions indicated that photofunctionalization accelerated and augmented the initial cell attachment, cell spreading, and subsequent differentiation of osteoblast lineage cells in the bone marrow to generate bone structures around the implant in vivo.

The technique of physicochemical surface modifications using UV photofunctionalization and protein coating to enhance biological properties was the highlight of our study. Additionally, greater protein adsorption, enhanced cellular attachment and spread, and differentiation of osteoblastic phenotype by virtue of UV photofunctionalization could ensure the progression of early bone-implant integration because of the increased potential for implant-bone contact. However, there were some limitations to our study with regard to clinical applications. First, although in vitro and in vivo studies demonstrated improvements in bone integration in an animal model when UV/FN surfaces were used, the actual mechanical anchorage capability of this surface remained unclear in humans. From a clinical perspective, it must be considered that a significant frictional force generally occurs between the implant surface and bone wall during implant placement. In the present study, the protein coating on the implant surface showed few side effects because of significant friction as we inserted the implant with a passive-fit. Briefly, the side effects due to friction were minimized with respect to the physical separation of the protein coated on the implant surface. In the actual clinical setting, many screw types are used in the dental field, and various types of implants are used in the orthopedic field. Therefore, depending on various applications or situations, it is necessary to conduct further animal experiments and determine how the protein coating is affected at the time of implant placement. Furthermore, even if the protein coated on the titanium surface is separated at the time of placement of an implant, the cells on the titanium surface will have positive action. Particularly, the growth factor proved in this research was effective. In the above situation, titanium plays a role as a delivery vehicle and coating device. We expect new and expanded clinical applications in these fields. In addition, these titanium surfaces should be evaluated for potential inflammatory adverse reactions or their susceptibility to microbial infections when used in experimental animal models. Finally, the clinical aspects of UV/FN implants must be further investigated to determine the optimal protein concentration, types of proteins to be used for coating, and intensity, wavelength, and exposure time of UV light to achieve optimal effects on the titanium surface. In conclusion, our study provided a new strategy for improving osteoconductivity by enhancing the bioactivity of titanium surfaces using UV photofunctionalization and protein coating. Therefore, this technology, combining the benefits of the mentioned processes, shows potential for various clinical applications, including dental and orthopedic surgical implants.

## 4. Materials and Methods

### 4.1. Titanium Surface Modifications

For in vitro and in vivo studies, commercially pure titanium disks (grade 2: 20 mm in diameter and 1 mm thick) and rod-shaped experimental titanium implants (grade 2: 1 mm in diameter and 2 mm length) were fabricated. The surfaces of the titanium discs and implants were treated by acid-etched using H_2_SO_4_ (Sigma-Aldrich, St. Louis, MO, USA) at 120 °C for 75 s. The prepared titanium disks were assigned to 4 groups. The control group (control) consisted of the acid-etched disks under the stated conditions. The UV-treated group (UV) consisted of acid-etched disks exposed to UV light on the etched surface for 12 min with a UV illuminator (TheraBeam^®^ SuperOsseo, Ushio Inc., Tokyo, Japan). Three hundred microliters of bovine plasma FN (100 µg/mL protein/ddH_2_O) (Sigma-Aldrich) solution was pipetted onto the acid-etched titanium surface of each FN-adsorbed group disk (FN); non-adherent protein was removed after 1-h incubation. The UV-treated and FN-adsorbed group disks (UV/FN) were first exposed to UV light for 12 min on the acid-etched surface; 300 µL of bovine plasma FN (Sigma-Aldrich, St. Louis, MO, USA) solution was added and incubated for 1 h, followed by the removal of non-adherent protein. The BMP-2-adsorbed group disks (BMP-2) were prepared by pipetting 300 µL of bovine plasma BMP-2 (800 ng/mL protein/ddH_2_O) (GenScript, Inc., Piscataway, NJ, USA) solution on the acid-etched titanium, incubating for 1 min, and removing the non-adherent protein. The elemental composition of the titanium surface was analyzed by XPS (Axis Ultra DLD spectrometer; Kratos Analytical, Ltd., Manchester, UK), which used mono-energetic soft X-rays (Al-Kα) under high vacuum conditions (6 × 10^−7^ Pa). Photofunctionalization was achieved by treating the implants with UV light for 12 min immediately before their use in the push-in test.

### 4.2. Bone Marrow-Derived Stromal Stem Cells Culture

Bone marrow-derived stromal stem cells isolated from the femurs of adult male Sprague Dawley rats were cultured in growth medium at 37 °C, 5% CO_2_ in a humidified incubator, as previously described (ARC #2005-175-41E, approved on January 30, 2018) [13]. Sub-confluent cells were detached by trypsin-EDTA and sub-cultured in new dishes. The culture medium was changed twice per week. Cells were seeded at a density of 3 × 10^4^ cells/cm^2^ on different treated titanium disks in polystyrene 12-well culture dishes.

### 4.3. Protein Adsorption Assay

Bovine plasma FN (Sigma-Aldrich) and bovine plasma BMP-2 (GenScript Inc.) were used as model proteins. Three hundred microliters of protein solution were pipetted onto and spread over a titanium disk. After different periods of incubation at 37 °C, non-adherent proteins were removed, and the initial whole solution was mixed with micro bicinchoninic acid (Thermo Fisher Scientific, Waltham, MA, USA) at 37 °C for 1 h [34]. The incubation time for the FN protein solution was 1 min and 1, 3, and 24 h, whereas that for the BMP-2 protein solution was 1 min, 1 h, 1 day, and 3 days. Protein was quantified using a spectrophotometer at 562 nm with a plate reader (SYNERGY H1, Biotek, Winooski, VT, USA).

### 4.4. Measurement of Initial Cell Attachment to the Titanium Surface

The initial attachment of cells to the different titanium surfaces was performed using WST-1 reagent (Roche Applied Science, Basel, Switzerland) and observed using confocal laser scanning microscopy (CLSM) (TCS SP5, Leica, Wetzlar, Germany) after 24 h of incubation. Following a previously established protocol [36], the absorption value was measured in a spectrophotometer at 450 nm with a plate reader (Biotek). For CLSM (TCS SP5, Leica), the cells were fixed with 10% formalin and stained with rhodamine-phalloidin dye (actin filament, red color; R415, Molecular Probes, Eugene, OR, USA) and vinculin (green color; ab11194, Abcam, Cambridge, UK) [37].

### 4.5. Analysis of Morphology and Morphometry of Osteoblastic Cells

Twenty-four hours after seeding, the cells were fixed in 10% formalin and stained with rhodamine-phalloidin (Molecular Probes). In addition, the cultures were immunostained with mouse anti-vinculin monoclonal antibody (Abcam), followed by fluorescein isothiocyanate-conjugated anti-mouse secondary antibodies (Abcam), to visualize the expression of vinculin. CLSM (TCS SP5, Leica) was used to examine cell morphology and cytoskeletal arrangement, as previously described [38]. The cell area, perimeter, and Feret’s diameter were quantified using the digitized images with a Java-based image-processing program (ImageJ ver.1.51j8, NIH, Bethesda, MD, USA).

### 4.6. Alkaline Phosphatase (ALP) Activity Assay

ALP activity was evaluated on day 7 using a colorimetric determination method. As previously described [39], the cultured cells were rinsed with ddH_2_O, and 250 µL of *p*-nitrophenyl phosphate was added, followed by incubation at 37 °C for 15 min. ALP activity was evaluated by measuring the released nitrophenol in the enzymatic reaction and determined at 405 nm using a plate reader (Biotek).

### 4.7. Implant Surgery

The implants were placed only in the left femurs of 10-week-old male Sprague-Dawley rats anesthetized by inhalation of 1–2% isoflurane. The diameter and length of the prepared implant were 1 mm and 2 mm, respectively. It was inserted with a passive-fit at 9 mm from the distal edge of the femur by drilling using a 0.8 mm round burr and reamers (#ISO 090 and 100), as previously described [37,40,41]. The implant placement site was enlarged to the same size as the implant diameter, and the implant was inserted with a passive-fit. Twenty-four animals (each group *n* = 6, implants *n* = 6) were included in the control, UV, FN, and UV/FN implant groups after a 2-week healing period. All experimental protocols using animals were reviewed and approved by the UCLA Animal Research Committee (ARC #2005-175-41E, approved on January 30, 2018) and followed the Public Health Service Policy for the Humane Care and Use of Laboratory Animals and the UCLA Animal Care and Use Training Manual guidelines.

### 4.8. Biomechanical Implant Push-In Test

The biomechanical implant push-in test was used to evaluate the mechanical strength of the bone-implant integration. Details of the procedure and method confirmation have been described previously [15,42]. A testing machine (Instron 5544 electromechanical testing system; Instron, Norwood, MA, USA), comprising a 2000-N load cell and push-in rod (diameter 0.8 mm), was used to load the implant vertically downward at a crosshead speed of 1 mm/min after a 2 weeks healing period. The push-in value was measured as the peak of the load-displacement curve, which was calibrated for the angle of the implant deviation.

### 4.9. Morphological and Chemical Profiling of Tissue around Titanium Implants

After the push-in test, the specimens of the femur with the implant were trimmed and positioned on a scanning electron microscope (SEM) (Nova 230 Nano SEM, FEI, Hillsboro, OR, USA) holder such that the implant surface was exposed. Remaining blood clots were removed by agitating in ddH_2_O; the specimens were soaked in agitated water for 1 h and dried at 55 °C in vacuum for 20 min (SpeedVac, Thermo Fisher Scientific), as previously described [43]. After carbon sputter-coating, the specimens were investigated by SEM and EDS (JSM-5900LV, Joel Ltd., Tokyo, Japan) to assess their morphology and chemical composition, respectively.

### 4.10. Statistical Analysis

All in vitro culture studies, except cell morphology, actin expression, and vinculin expression analysis, were performed in technical triplicate (*n* = 3). Cell morphology, actin expression, and vinculin expression analyses were performed by calculating eight osteoblasts in each field. All statistical analyses were performed using GraphPad Prism version 6 (GraphPad, Inc., San Diego, CA, USA), and the results are expressed as the means ± standard deviation. Two-way ANOVA was used to examine the effect of healing time. When appropriate, a post hoc Bonferroni test was used to perform multiple comparison tests. If data were available at only one time-point, one-way ANOVA was used to determine the significantly different surface groups. Where appropriate, Tukey’s HSD test was used as a post hoc analysis. In addition, Welch’s *t*-test was performed to compare the control and UV according to protein adsorption and cell attachment. The *p*-values less than 0.05 were considered as statistically significant.

## 5. Conclusions

UV-pre-treatment of titanium accelerated and enhanced the protein adsorption on its surface and resulted in increased attachment and function of osteoblasts. The in vivo biomechanical strength of bone and titanium integration was greater when the implants were UV-treated and protein-adsorbed than when the implants were only protein-adsorbed. From a clinical perspective, UV-mediated protein coating on titanium surfaces is a promising strategy for improving bone-implant interaction and accelerating the healing time.

## Abbreviations

ALP	alkaline phosphatase
BMP-2	bone morphogenetic protein-2
CLSM	confocal laser scanning microscopy
FN	fibronectin
ddH_2_O	double-distilled H_2_O
EDS	energy-dispersive X-ray spectroscopy
RGD	arginine-glycine-aspartate
SEM	scanning electron microscopy
UV	ultraviolet
WST	water-soluble tetrazolium salts
XPS	x-ray photoelectron spectroscopy
